# Solution NMR Structure and Histone Binding of the PHD Domain of Human MLL5

**DOI:** 10.1371/journal.pone.0077020

**Published:** 2013-10-09

**Authors:** Alexander Lemak, Adelinda Yee, Hong Wu, Damian Yap, Hong Zeng, Ludmila Dombrovski, Scott Houliston, Samuel Aparicio, Cheryl H. Arrowsmith

**Affiliations:** 1 Northeast Structural Genomics Consortium and Ontario Cancer Institute, University Health Network, Toronto, Ontario, Canada; 2 Structural Genomics Consortium, University of Toronto, Ontario, Canada; 3 Department of Molecular Oncology, BC Cancer Agency, Vancouver, British Columbia, Canada; 4 Structural Genomics Consortium, Northeast Structural Genomics Consortium, Ontario, Canada; 5 Cancer Institute and Department of Medical Biophysics, University of Toronto, Ontario, Canada; George Washington University, United States of America

## Abstract

Mixed Lineage Leukemia 5 (MLL5) is a histone methyltransferase that plays a key role in hematopoiesis, spermatogenesis and cell cycle progression. In addition to its catalytic domain, MLL5 contains a PHD finger domain, a protein module that is often involved in binding to the N-terminus of histone H3. Here we report the NMR solution structure of the MLL5 PHD domain showing a variant of the canonical PHD fold that combines conserved H3 binding features from several classes of other PHD domains (including an aromatic cage) along with a novel C-terminal α-helix, not previously seen. We further demonstrate that the PHD domain binds with similar affinity to histone H3 tail peptides di- and tri-methylated at lysine 4 (H3K4me2 and H3K4me3), the former being the putative product of the MLL5 catalytic reaction. This work establishes the PHD domain of MLL5 as a bone fide ‘reader’ domain of H3K4 methyl marks suggesting that it may guide the spreading or further methylation of this site on chromatin.

## Introduction

Post translational modifications of histones are a key epigenetic mechanism used to regulate gene transcription, chromatin condensation, DNA damage sensing and repair. Key among these modifications are protein lysine acetylation and methylation. These modifications are “written” or “erased” by chromatin-associated proteins that have the specific catalytic activities. These modifications are in turn recognized by “reader” domain(s) of proteins that are recruited to the chromatin. Better known examples of reader domains include chromodomain [1], [2], bromodomain [3], MBT domain [4], TUDOR domain [5], WD40 domain [6], PWWP [7], and PHD finger [8], [9], [10].

PHD (Plant HomeoDomain) fingers are small modules with conserved cysteines and histidine coordinating 2 zinc ions in a canonical Cys4-His-Cys3 mode. Based on the Pfam protein family classification, the PHD finger is found in over 100 proteins in the human genome. Proteins with PHD fingers are mostly nuclear [10] and often involved in chromatin remodelling. PHD fingers studied so far recognize several different histone trimethyllysine marks [11], [12] as well as unmodified histone H3 N-terminus [13], [14], and possibly acetyllysine [15].

Mixed Lineage Leukemia 5 (MLL5) is a SET domain methyltransferase and contains a single PHD finger followed by a catalytic SET domain. MLL5 protein localizes to distinct nuclear foci, but this activity was not affected by deletion of either the PHD domain or the SET domain [16]. Overexpression of MLL5 prevented cell cycle progression into S phase by associating with cell cycle regulatory elements impairing its activity [16]. Phosphorylation of the C-terminus of the SET domain of MLL5 is required for mitotic progression, suggesting a role for histone methylation [17]. Immunoprecipitation and *in-vitro* pull down experiments showed that MLL5 interacts with borealin, a subunit of the chromosome passenger complex, stabilizing the complex [18]. MLL5 is also reported to bind with tetrameric p53 via p53's DNA binding domain [19]. MLL5 is a component of a complex associated with retinoic acid receptor that requires GlcNAcylation of its SET domain in order to activate its histone lysine methyltransferase activity [20]. Knockout mice studies showed that murine MLL5 is required in normal hematopoiesis [21], [22], [23] as well as maturation of spermatozoa [24]. However, except for nuclear foci formation, the role of the PHD domain in these activities has not been delineated.

We report the solution NMR structure of the PHD domain of MLL5 and confirm its binding to histone H3 peptides di- and tri-methylated at lysine 4 (H3K4me2/3). Importantly, the latter, but not the former is thought to be the product of the methyltransferase activity of the MLL5 [25]. We propose a binding mechanism based on the newly determined structure and its comparison with other PHD domains combined with biophysical interaction data with histone peptides. This data supports the growing observation that many histone modifying enzymes have evolved specialized ‘reader’ domains that recognize the reaction product of their catalytic domains, which may help with spreading of the respective histone mark along chromatin, and/or the further methylation of this mark by a separate methyltransferase.

## Results and Discussion

### Solution Structure of MLL5_PHD_ finger

**Figure 1:**
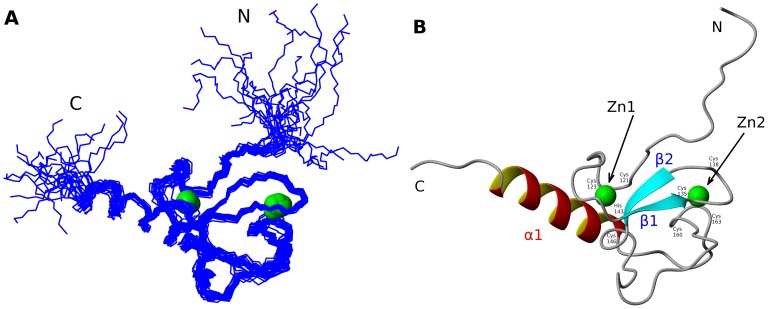
Solution structure of PHD domain of human MLL5 (A) The backbone trace of the 20 structures comprising the lowest energy NMR ensemble is shown in blue (B) Ribbon diagram of a representative structure of the NMR ensemble. Zinc atoms are shown as green spheres.

**Figure 2:**
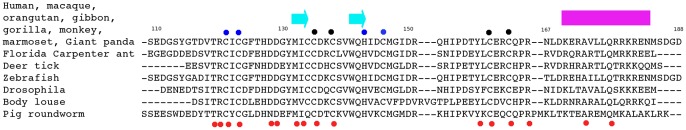
Multiple sequence alignment of PHD domains of MLL5 in various organisms. Conserved residues are marked with red circles at the bottom [29]. Organism of origin is shown on the left-hand-side of each sequence. Secondary structure elements of the PHD domain are shown above its sequence for clarity (α-helix as cylinder and β-strands as arrows). The residues coordinating Zn1 and Zn2 atoms are marked with blue and black dots at the top, respectively. Homologous domains are identified using protein blast against non-redundant protein database (http://blast.ncbi.nlm.nih.gov/blast.cgi). multiple sequence alignment is performed using clustalw2 (www.ebi.ac.uk/tools/clustalw2).

Using an 80 residues protein construct spanning the PHD domain of MLL5 (Ser109-Asp188) we determined its solution structure by NMR spectroscopy (Figure 1). The N-terminal region of the domain, residues 109–117, appears to be disordered in solution, while the structure of the rest of the domain (residues 118–183) is well defined with a backbone r.m.s.d of 0.86+0.17 Å (see Table 1). It comprises two small antiparallel β-strands, β1 and β2 (residues 132–134, and residues 141–143, respectively), one α-helix, α1 (residues 170–183), and three long loops stabilized by two zinc-binding clusters. Similar to the other structurally characterized PHD domains, MLL5_PHD_ domain binds two Zn^2+^ ions in a cross-braced fashion. Zn1 atom is coordinated by Cys121, Cys123, His143, and Cys146 while Zn2 atom is coordinated by four cysteine residues Cys135, Cys138, Cys160 and Cys163, respectively (see Figures 1a and b). All Zn-coordinated residues and the key residues from the hydrophobic core are highly conserved among homologous MLL5 PHD domains (Figure 2).

**Table 1:** NMR data and refinement statistics.

NMR distance and dihedral constraints	
***Distance restraints:***	
Total NOE	1476
Intra-residual	388
Sequential (|i–j| = 1)	378
Medium-range (2≤ |i–j| ≤4)	281
Long-range (|i–j| >4)	429
Hydrogen bonds	14
Zinc-ligand distance restraints	23
***Dihedral Angle restraints:***	
φ	61
ψ	61

Structure statistics	
***Restraints Violations***	
Distance constraints (mean ± s.d.)(Å)	0.0182±0.0025
Dihedral angle constraints (mean ± s.d.)(°)	0.749±0.18
Max. distance constraints violation (Å)	0.51
Max. dihedral angle violation (°)	6.8
***Deviations from idealized geometry***	
Bond lengths (Å)	0.0136±0.0002
Bond angles (°)	0.93±0.024
Impropers (°)	1.86±0.15
***Ramachandran plot*** a	
Most favored region (%)	85.9
Allowed (%)	14.0
Generous (%)	0.1
Disallowed (%)	0.0
***Average pairwise r.m.s.d***.^b^ (Å)	
Heavy	1.57±0.23
Backbone	0.86±0.17
**Global quality scores** c	
RawZ-score	
Procheck (phi-psi)a	−0.29–0.83
Procheck (all)a	−0.23–1.36
MolProbity clash	14.73–1.00
**RPF scores** d	
Recall	0.94
Precision	0.93
DP-score	0.768

a Values calculated for the ordered regions, as reported by PSVS [26]: residues 118–183.

b r.m.s.d calculated for residues 118–183.

c Calculated by PSVS.

d RPF scores [27] reflecting the goodness-of-fit of the structural ensemble to the NMR data.

**Figure 3:**
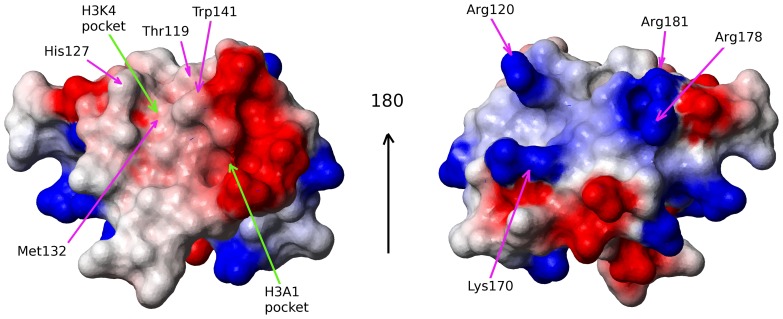
Molecular surface representation of the PHD domain shown from two points of view. The surface is colored according to electrostatic potential (red for negative charges and blue for positive charges). The orientation of the domain is as in Figure 1. MOLMOL [28] was used to create this figure.

Electrostatic surface representation of the MLL5_PHD_ domain is shown in Figure 3. One can see an extended putative H3 peptide binding surface groove typical for PHD fingers [8]. Namely, there are two adjacent hydrophobic pockets (presumably for H3 Lys4 and H3 Ala1 binding, respectively) divided by a tryptophan (Trp141). Trp141 occupies the conserved ‘position I’ of the H3K4me2/3-binding aromatic cage commonly observed in PHD domains [8]. In the majority of the NMR ensemble models, His127 forms the opposite side of a minimal aromatic cage, and is complemented by Thr119 and Met132 which complete a hydrophobic pocket that is likely to bind di- or tri-methlysine (Figure 3). During preparation of this manuscript, a crystal structure of the MLL5_PHD_ in complex with H3K4me3 was published and suggests that His127 is replaced by Asp128 [30].

**Figure 4:**
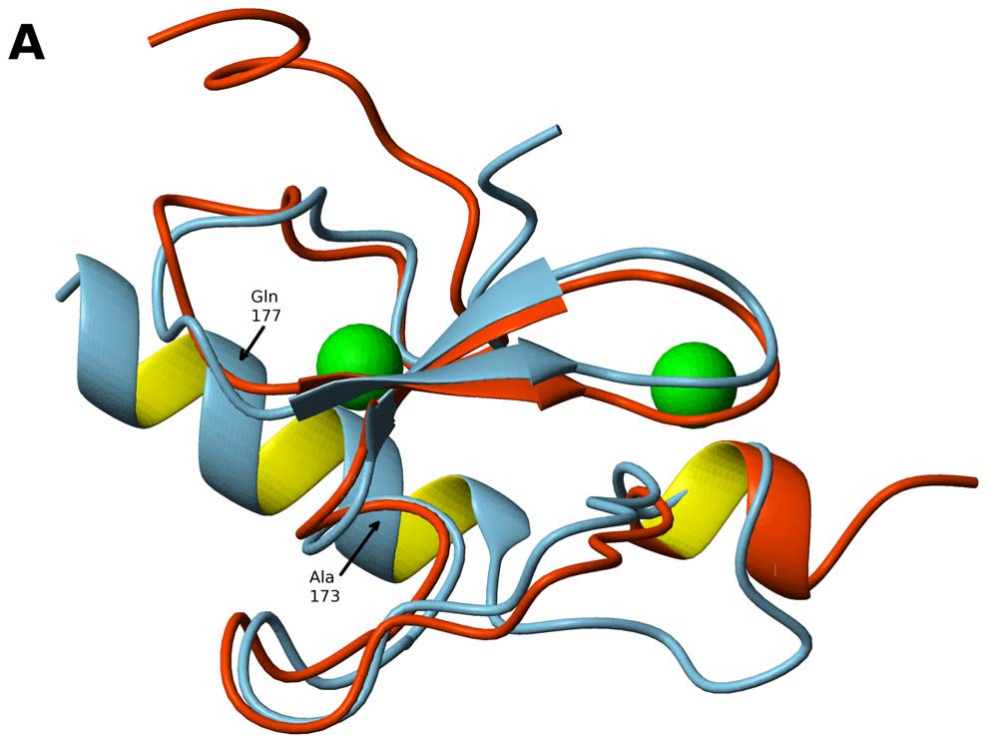
Comparison of PHD domains of MLL5 and PHF13 proteins. Ribbon representation of the domains with superimposed backbone, MLL5_PHD_ in blue and unbound PHF13_PHD_ (PDB ID 3O70) in orange.

A novel feature of the MLL5_PHD_ domain is a long α helix(helix α1) not present in any other published PHD structures and formed by a C-terminal sequence unique to MLL5 and its homologs (Figures 1,2,4). This helix folds onto the canonical PHD finger on the opposite side from the peptide binding groove via hydrophobic interactions involving two highly conserved residues, Ala173 and Gln177 (Figure 2, 4A). The solvent exposed face of helix α1 consists of positively charge residues Lys170, Arg178, and Arg181 that are poorly conserved in the homologous MLL5 proteins (see Figure 3). This suggests that the role of helix α1 may be to act as a ‘structural brace’ for the PHD domain, as opposed to forming a new interaction surface on the solvent exposed face of the helix.

To determine structural homologs of the PHD domain of MLL5 we used the DALI server [31]. Many PHD domains with significant similarity (Z-score >4.0) were detected. For example, the PHD domain of human BPTF (PDB ID 3QZV, 2FSA) has 38% sequence identity with MLL5_PHD_ and Z-score of 4.7. The best match to MLL5 was human PHD finger protein 13 (PHF13) from (PDB ID 3O70; Z-score 5.7) which has only 28% sequence identity with MLL5_PHD_. Nevertheless these two PHD domains can be structurally aligned with a backbone r.m.s.d. of 1.9 Å over 47 residues (see Figure 4a). Comparison of the putative methyl lysine binding pocket of the MLL5_PHD_ with that of the peptide-bound PHF13_PHD_ (PDB ID 3O7A) showed that MLL5_PHD_ is likely to bind H3K4me3 in the same manner as PHF13_PHD_.

### Histone Recognition of MLL5

MLL5 is reported to bind directly to chromatin at the cell cycle regulated element [32]. However, MLL5 lacks an obvious DNA binding motif. Furthermore, MLL5 has been identified as GlcNAcylation-dependent H3K4 methyltransferase component of the RARA complex [20]. Since PHD fingers are known to bind histone tails [11] and our structure shows a potential histone peptide binding pocket conserved among several complexes between PHD fingers and histone tails with differing lysine modifications have been reported in the PDB, we hypothesized that MLL5_PHD_ also binds methylated histone H3 tails.

**Figure 5:**
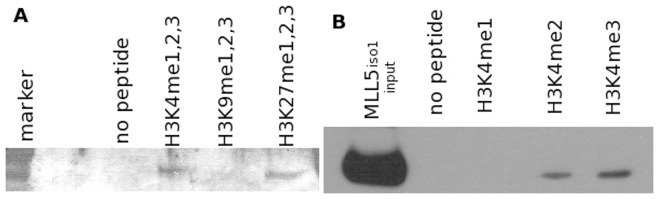
MLL5_iso1_ and histone H3 peptides complexes. Purified MLL5_iso1_ incubated with biotinylated H3 peptides and complex was pulled down using streptavidin agarose beads and detected using anti-MLL5 antibody. (A) Pull down assay using H3 peptides with methylation at different lysine sites. (B) Pull down assay using H3 peptides with varying degrees of methylation at the K4 site.

We first performed an initial *in-vitro* peptide binding assay on his-tagged MLL5_iso1_ equivalent to isoform 1 that contains both PHD and SET domains (residue 1 to 609). A mixture of biotinylated H3 peptides with various degrees of methylation at different lysine sites was incubated with purified MLL5_iso1_. MLL5_iso1_/H3 peptide complexes were pulled down using streptavidin-agarose beads and the presence of the complex detected using an anti-MLL5 antibody (Figure 5a). The streptavidin pull down assay showed that MLL5_iso1_ binds to methylated H3K4 and H3K27 peptides but not to H3K9 peptides. To further deconvolute the binding to H3K4, the same experiment was repeated with biotinylated H3K4 peptides with differing degrees of methylation (Figure 5b). This showed that H3K4 MLL5_iso1_ binds to both H3K4me2 and H3K4me3 peptides. We did not detect any binding of MLL5_iso1_ to monomethylated H3K4 peptides.

To determine if the same binding mode applies to the PHD finger alone, a peptide array of different histone sequences with differing lengths and lysine/arginine modifications was synthesized. Purified his-tagged MLL5_PHD_ was incubated with the membrane and detected using anti-HIS antibody (supplementary Figures S1 and S2). The peptide array confirmed that MLL5_PHD_ consistently binds to H3K4me3. This binding was not abrogated by methylation on the R2 or R8 positions, nor with phosphorylation on the S10 position. Phosphorylation at the T3 position appeared to diminish binding to the H3K4me3 spot, as did deletion of the first 3 residues, suggesting an important contribution from residues 1–3 in the interaction.

**Figure 6:**
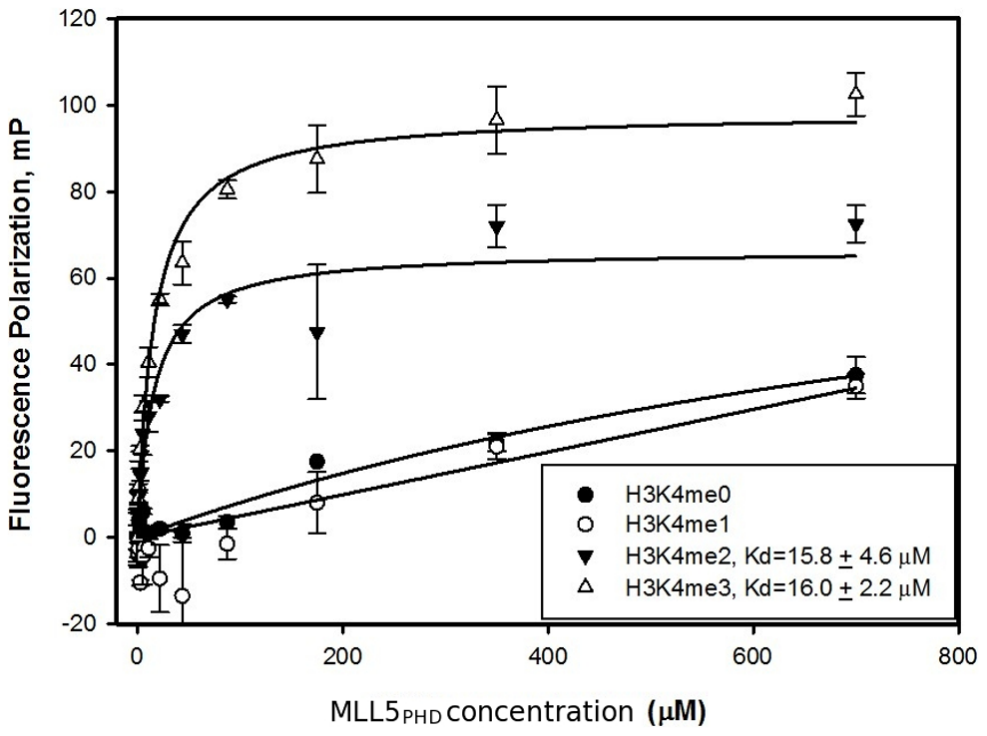
MLL5_PHD_ binding to H3K4 peptides as detected by fluorescence polarization.

The peptide array results did not show reproducible binding of the MLL5_PHD_ to any other acetyl- or methyl-lysine marks within H3 peptides, including H3K9 and H3K27. This suggests that the potential H3K27me binding activity observed for MLL5_iso1_ must reside in regions of the protein other than the PHD domain. Since immobilized peptide arrays are semiquantitative at best, and prone to false positive and negative results [33], we sought to confirm these results with more quantitative analyses using free components in solution. A fluorescence anisotropy assay using H3K4 peptides labelled with fluorescein at the C-terminus enabled measurement of the equilibrium dissociation constants (Figure 6). Consistent with our peptide array result, both H3K4me2 and H3K4me3 peptide bind to MLL5_PHD_ with a similar dissociation constant of ∼16 uM.

**Figure 7:**
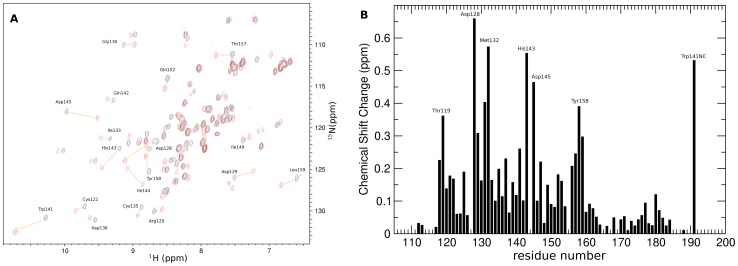
H3K4me3 peptide titration of MLL5_PHD_. (A) ^15^N-HSQC spectra of ^15^N-labelled MLL5_PHD_ before (black) and after (red) addition of H3K4me3 peptide. (B) Normalized chemical shift changes upon H3K4me3 binding. The normalized chemical shift perturbations for backbone ^15^N and ^1^H_N_ resonances were calculated using the equation 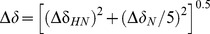, were Δδ is the change in chemical shift in ppm.

**Figure 8:**
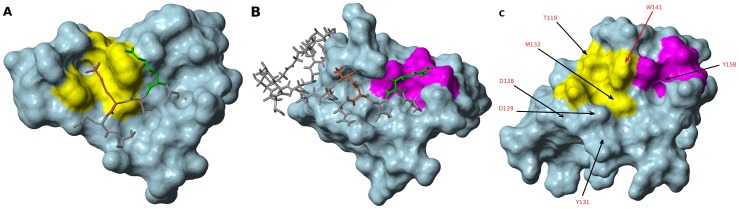
Surface comparison between PHD domains of PHF13_PHD_, [PDB ID: 3O7A] (A, AIRE_PHD_, [PDB ID 2KFT] (B) and that of MLL5_PHD_ (C). Peptide K4 is in orange stick, R2 in green stick. (A) PHF13's Trp255, Phe241, Met246, Thr234 are highlighted in yellow. (B) AIRE's Cys310, Asp312, Thr334, Trp335 are highlighted in magenta (C) MLL5's Trp141, Met132, His127, Thr119 are highlighted in yellow and Cys134, Asp136, Thr157, Tyr158 are highlighted in magenta. Residues for which chemical shifts have changed by more than 0.3 ppm are indicated.

The binding of H3K4me3 peptide to MLL5_PHD_ was further confirmed by ^15^N-HSQC NMR titration revealing significant changes in the NMR spectrum of ^15^N-labelled MLL5_PHD_ upon increasing amounts of H3K4me3 peptide (Figure 7a). The residues involved in peptide binding can be inferred from the chemical shift changes (Figure 7b) and map to the conserved histone peptide binding region described above (Figure 8c). The residues affected the most by this binding involved Thr119, Asp128, Met132, His143, Asp145, Tyr139 and Trp141. This includes the key residues of the aromatic cage with the exception of His127, whose ^15^NH resonance is not visible in the HSQC reference spectrum.

The strong chemical shift changes in the aromatic cage combined with the structural similarity between MLL5_PHD_ and PHF13_PHD_ domains suggest a similar H3 peptide binding mode (Figure 4b, 8a). On the other hand, the surface of the putative H3R2 binding site is different between these two proteins. MLL5_PHD_ has a negatively charged pocket comprising a putative H3R2 binding site that is absent in the case of PHF13_PHD_ (see Figure 8a). The structure of the complex of the PHF13_PHD_ and H3K4me3 peptide shows that the side chains of H3R2 is not docked tightly to the surface of the PHD domain which is in agreement with the absence of a corresponding H3R2 binding pocket. The putative H3R2 binding pocket on the surface of MLL5_PHD_ domain is formed by the well conserved residues Cys134, Asp136, Tyr158, and Thr157. A very similar groove, formed with the same type of residues can be found in the structure of the complex of human AIRE_PHD1_ domain with the unmodified H3 peptide (Figure 8b) [13], [34]. Structural alignment of MLL5_PHD_ and AIRE_PHD1_ indicate that key H3R2 binding residues of AIRE_PHD1_ Cys310, Asp312, Trp335, and Thr157 superimpose well with the Cys134, Asp136, Tyr158, and Thr157 of MLL5_PHD_ domain, respectively. These residues in MLL5_PHD_ show modest changes in chemical shift upon peptide titration, consistent with a modest contribution to binding affinity and a tolerance for methylation of Arg2 in binding to peptide arrays. Finally the key residues of the H3A1 binding site (Tyr158) also shows large chemical shift changes upon H3 peptide binding (see Figure 8). Taken together our structural and biochemical data support the role of MLL5_PHD_ as a specific ‘reader’ domain of H3K4me2/3 marks.

### Conclusion

We have determined the solution structure of the PHD finger of MLL5 and observed very similar structural features compared to other PHD fingers. It was reported that in MLL5 knockdown cells, H3K4 methylation at the cell cycle regulated element is reduced [16], and H3K4 trimethylation levels are also reduced at E2F1 target promoters [25]. The preferential binding of the PHD domain to di- and trimethylated H3K4 is one of the most frequently observed examples of a growing number for “reader domains” that reside within larger enzymes or enzyme complexes that ‘write’ the same mark. It has been proposed that a potential role for such a function may be to help facilitate spreading of the mark along chromatin by the reader domain binding to the product of the catalytic reaction, enabling the enzyme to then modify a neighboring histone/nucleosome [35], [36]. Our data suggest that the PHD domain of MLL5 may serve such a role in its modification of genomic loci with the H3K4me2/3 mark.

## Materials and Methods

### Protein Expression and Purification

The PHD finger of MLL5 (residue 109–188) was inserted into a pET28a-MHL vector (GenBank ID: EF456735) via ligase-independent cloning. The recombinant protein was expressed in BL21 (DE3) Codon plus RIL (Stratagene). For proteins used for peptide array, cells were grown in rich Terrific Broth (Sigma); for proteins used for NMR structure determination, cells were grown in minimal media containing ^13^C-glucose and ^15^N-NH_4_Cl as the sole carbon and nitrogen source, respectively. The cells were grown at 37°C and induced with IPTG when cells reach the mid-log phase of growth for another 12 hours.

For unlabelled MLL5 protein, the cell pellet was resuspended in phosphate buffered saline containing 250 mM NaCl, 2 mM 2 mM β-mercaptoethanol, 5% glycerol, 0.1% CHAPS, 1 mM PMSF. Cells were disrupted by passing through Microfluidizer (Microfluidics Corp.) at 20,000 psi. After high speed centrifugation, the lysate was loaded onto 5 ml HiTrap column (GE Healthcare), charged with Ni^2+^. The column was washed with 10 CV of 20 mM Tris-HCl pH 8.0, containing 250 mM NaCl, 50 mM imidazole, 5% glycerol, and the protein was eluted with elution buffer (20 mM Tris-HCl pH 8.0, 250 mM NaCl, 250 mM imidazole, 5% glycerol). The protein was loaded on Superdex200 column (26×60) (GE Healthcare), equilibrated with 20 mM Tris-HCl buffer, pH 8.0, and 150 mM NaCl. The protein was further purified to homogeneity by ion-exchange chromatography on Source 30Q column (10×10) (GE Healthcare), equilibrated with buffer 20 mM Tris-HCl, pH 8.0, and eluted with linear gradient of NaCl up to 500 mM concentration (20 column volumes).

For ^13^C and ^15^N labeled protein used for NMR studies, cells were harvested by centrifugation and resuspended in lysis buffer (10 mM tris, pH 8.5, 15 mM imidazole, 500 mM NaCl, 10 uM ZnSO_4_). The cells were lysed by sonication and cell debris were removed by centrifugation at 12000 rpm for 20 min at 4C. The supernatant was bound to Ni-NTA beads and washed extensively with washing buffer (10 mM tris, pH 8.5, 30 mM imidazole, 500 mM NaCl, 10 uM ZnSO_4_). Target protein was eluted with elution buffer (10 mM tris, pH 8.5, 500 mM imidazole, 500 mM NaCl, 10 uM ZnSO_4_). After elution, benzamidine and DTT was added to a final concentration of 1 mM each.

### NMR Structure Determination

NMR spectra were recorded at 25°C on Bruker Avance 600 MHz or 800 MHz spectrometers equipped with cryoprobes. All 3D spectra employed non-uniformly sampling scheme in the indirect dimensions and were reconstructed by multi-dimensional decomposition software MDDNMR [37] interfaced with MDDGUI [38] and NMRPipe [39]. The assignments of ^1^H, ^15^N and ^13^C resonances were obtained by an ABACUS [40] approach using the following experiments: HNCO, CBCA(CO)NH, HBHA(CO)NH, HNCA, (H)CCH-TOCSY and H(C)CH-TOCSY. Distance restraints for structure calculations were derived from cross-peaks in ^15^N-edited NOESY-HSQC (τm  = 100 ms), ^13^C-edited aliphatic and aromatic NOESY-HSQC in H_2_O (τm  = 100 ms) respectively. Peak picking was performed manually using Sparky [41]. The restraints for backbone φ and ψ torsion angles were derived from chemical shifts of backbone atoms using TALOS [42]. Automated NOE assignment and structure calculations were performed using CYANA (version 2.1) [43]. A total of 93% of NOESY peaks were assigned after seven iterative cycles of automated structure calculation and NOE assignment. The final 20 lowest-energy structures were refined with the CNS [44] package by performing a short constrained molecular dynamics simulation in explicit solvent [45]. Resulting structures were analyzed using MOLMOL [28], PROCHEK [46], MOLProbity [47], and PSVS validation software [26]. The final refined ensemble of 20 structures and resonance assignments for MLL5_PHD_ domain were deposited into the Protein Data Bank (PDB ID, 2LV9) and BioMagRes DB (BMRB accession number 18559), respectively.

### Streptavidin Pull Down Assay

Human MLL5 ORF v3.1 was shuttled from pDONR223 into pDEST17 which expressed N-terminal 6× His Tag fusion MLL5 protein from the T7 promoter. Purified his-tagged MLL5_iso1_ was stored in buffer (30 mM imidazol, 116 mM NaCl, 25 mM Tris-HCl pH 7.5, 3 mM KCl) and then an aliquot was incubated with 0.5 µg biotinylated histone H3 peptides (residues 1–21 or 21–44, Upstate) in binding and washing buffer (25 mM Tris, pH 7.5, 120 mM NaCl, 3 mM KCl and 0.05% (v/v) Nonidet P-40) for 4 h at 4°C. Streptavidin-Sepharose 4B beads (Upstate 16–126) incubated with 6× His-hMLL5: peptide binding reactions overnight at 4°C. Beads: complexes were then washed three separate times each in 1 ml binding and washing buffer at 4°C and heated to 90°C for 7 min with NuPage Sample buffer (Invitrogen) with reducing agent. The lysates were loaded on a 4–12% NuPage gradient gel (pre-cast from Invitrogen), run at 200 V for 40 min and blotted on nitrocellulose membranes using the iBlot semi-dry transfer system (Invitrogen; program P3 for 7 min). The membranes were probed with anti-MLL5 antibodies (pAb 31994 Custom made antibody in serum from Rabbit #9762) and 1/3000 anti-rabbit-HRP and visualized using the Immobilon Western Chemiluminescent HRP System (Millipore).

### Peptide Array

Peptide arrays were synthesized using Intavis. The array was blocked at 4^°^C overnight with 5% skimmed milk in PBS-T (50 mM Na_3_PO_4_ pH  = 7.5, 110 mM NaCl, 0.05% Tween 20), and washed three times with PBS-T. For identifying the binding site of each peptide within MLL5_PHD_, the MLL5 PHD protein was diluted in 1% milk in PBS-T to 1 µM. The protein was incubated with the membrane overnight at 4°C. The array was washed three times with PBS-T. Protein was detected using HRP-conjugated anti-His antibody (Novagen).

### Fluorescence Anisotropy Binding Studies

Fluorescence polarization assays were performed in 384-well plates, using the Synergy 2 microplate reader from BioTek. All the peptides were synthesized and purified by Tufts University Core Services (Boston, MA, U.S.A.), with the N-terminus labeled with fluorescein. Binding assays were performed in a 10 μL volume at a constant labeled peptide concentration (40 nM), by titrating the MLL5_PHD_ domain (at concentrations ranging from low to high micromolar) into 20 mM Tris-HCl buffer (pH 7.5), containing 50 mM NaCl, 0.01% Triton X-100. The data points were fitted to ligand binding function using Sigma Plot software to determine the *K*_d_ values.

## Supporting Information

Figure S1 **H3 histone tail peptide array bound with his-tagged MLL5_PHD_.** Protein was detected using anti-His antibody. Left panel showed the no protein control, only the poly-His spot was detected by the anti-His antibody. Right panel showed the peptide spots where MLL5_PHD_ proteins were bound. The letters on each grid highlight which residues on the H3 histone tail was modified. Actual peptide sequence on the array is shown in Supplementary Figure S2.
(JPG)

Figure S2 **Peptide sequence of the peptide array in Figure S1.** Grid location refers to Figure S1a.
(JPG)

## References

[1] Jones DO, Cowell IG, Singh PB (2000) Mammalian chromodomain proteins: their role in genome organisation and expression. BioEssays 22: 124–137. 10655032 10.1002/(SICI)1521-1878(200002)22:2<124::AID-BIES4>3.0.CO;2-E

[2] Yap KL, Zhou M (2011) Structure and Mechanisms of Lysine Methylation Recognition by the Chromodomain in Gene Transcription. Biochemistry 50: 1966–1980. 21288002 10.1021/bi101885m PMC3062707

[3] Zeng L, Zhou M (2002) Bromodomain: an acetyl-lysine binding domain. FEBS Letters 513: 124–128. 11911891 10.1016/s0014-5793(01)03309-9

[4] Bonasio R, Lecona E, Reinberg D (2010) MBT domain proteins in development and disease. Seminars in Cell & Developmental Biology 21: 221–230. 19778625 10.1016/j.semcdb.2009.09.010 PMC3772645

[5] Botuyan MV, Lee J, Ward IM, Kim J, Thompson JR, et al (2006) Structural Basis for the Methylation State-Specific Recognition of Histone H4-K20 by 53BP1 and Crb2 in DNA Repair. Cell 127: 1361–1373. 17190600 10.1016/j.cell.2006.10.043 PMC1804291

[6] Xu C, Min J (2011) Structure and function of WD40 domain proteins. Protein Cell 2: 202–214. 21468892 10.1007/s13238-011-1018-1 PMC4875305

[7] Wu H, Zeng H, Lam R, Tempel W, Amaya MF, et al (2011) Structural and Histone Binding Ability Characterizations of Human PWWP Domains. PLoS One 6: e18919. 21720545 10.1371/journal.pone.0018919 PMC3119473

[8] Sanchez R, Zhou M (2011) The PHD Finger: A Versatile Epigenome Reader. Trends Biochem Sci 36: 364–372. 21514168 10.1016/j.tibs.2011.03.005 PMC3130114

[9] Aasland R, Gibson TJ, Stewart AF (1995) The PHD finger: implications for chromatin-mediated transcriptional regulation. Trends Biochem Sci 20: 56–59. 7701562 10.1016/s0968-0004(00)88957-4

[10] Bienz M (2006) The PHD finger, a nuclear protein-interaction domain. Trends Biochem Sci 31: 35–40. 16297627 10.1016/j.tibs.2005.11.001

[11] Musselman CA, Kutateladze TG (2009) PHD Fingers: Epigenetic Effectors and Potential Drug Targets. Mol Interv 9: 314–323. 20048137 10.1124/mi.9.6.7 PMC2861807

[12] Sims RJ 3rd, Millhouse S, Chen CF, Lewis BA, Erdjument-Bromage H, et al (2007) Recognition of trimethylated histone H3 lysine 4 facilitates the recruitment of transcription post initiation factors and pre-mRNA splicing. Mol Cell 28: 665–676. 18042460 10.1016/j.molcel.2007.11.010 PMC2276655

[13] Chignola F, Gaetani M, Rebane A, Org T, Mollica L, et al (2009) The solution structure of the first PHD finger of autoimmune regulator in complex with non-modified histone H3 tail reveals the antagonistic role of H3R2 methylation. Nucleic Acids Res 37: 2951–2961. 19293276 10.1093/nar/gkp166 PMC2685098

[14] Rajakumara E, Wang Z, Ma H, Hu L, Chen H, et al (2011) PHD finger recognition of unmodified histone H3R2 links UHRF1 to regulation of euchromatic gene expression. Mol Cell 43: 275–284. 21777816 10.1016/j.molcel.2011.07.006 PMC4691841

[15] Zeng L, Zhang Q, Li S, Plotnikov AN, Walsh MJ, et al (2010) Mechanism and Regulation of Acetylated Histone Binding by the Tandem PHD Finger of DPF3b. Nature 466: 258–262. 20613843 10.1038/nature09139 PMC2901902

[16] Deng L, Chiu I, Strominger JL (2004) MLL 5 protein forms intranuclear foci, and overexpression inhibits cell cycle progression. Proc Natl Acad Sci U S A 101: 757–762. 14718661 10.1073/pnas.2036345100 PMC321754

[17] Liu J, Wang XN, Cheng F, Liou Y, Deng L (2010) Phosphorylation of Mixed Lineage Leukemia 5 by Cdc2 Affects Its Cellular Distribution and Is Required for Mitotic Entry. J Bio Chem 285: 20904 –20914. 10.1074/jbc.M109.098558 PMC2898323 20439461

[18] Liu J, Cheng F, Deng L (2012) MLL5 maintains genomic integrity by regulating the stability of the chromosomal passenger complex through a functional interaction with Borealin. Journal of Cell Science 125: 4676–4685. 22797924 10.1242/jcs.110411 PMC3500868

[19] Cheng F, Liu J, Teh C, Chong SW, Korzh V, et al (2011) Camptothecin-induced downregulation of MLL5 contributes to the activation of tumor suppressor p53. Oncogene 30: 3599–3611. 21423215 10.1038/onc.2011.71

[20] Fujiki R, Chikanishi T, Hashiba W, Ito H, Takada I, et al (2009) GlcNAcylation of a histone methyltransferase in retinoic-acid-induced granulopoiesis. Nature 459: 455–459. 19377461 10.1038/nature07954

[21] Heuser M, Yap DB, Leung M, de Algara TR, Tafech A, et al (2009) Loss of MLL5 results in pleiotropic hematopoietic defects, reduced neutrophil immune function, and extreme sensitivity to DNA demethylation. Blood 113: 1432–1443. 18854576 10.1182/blood-2008-06-162263

[22] Zhang Y, Wong J, Klinger M, Tran MT, Shannon KM, et al (2009) MLL5 contributes to hematopoietic stem cell fitness and homeostasis. Blood 113: 1455–1463. 18818388 10.1182/blood-2008-05-159905 PMC2644073

[23] Madan V, Madan B, Brykczynska U, Zilbermann F, Hogeveen K, et al (2009) Impaired function of primitive hematopoietic cells in mice lacking the Mixed-Lineage-Leukemia homolog MLL5. Blood 113: 1444–1454. 18952892 10.1182/blood-2008-02-142638

[24] Yap DB, Walker DC, Prentice LM, McKinney S, Turashvili G, et al (2011) Mll5 Is Required for Normal Spermatogenesis. PLoS ONE 6: e27127. 22069496 10.1371/journal.pone.0027127 PMC3206077

[25] Zhou P, Wang, Z Yuan, X Zhou, C Liu, L Wan, et al (2013) Mixed Lineage Leukemia 5 (MLL5) Regulates Cell Cycle Progression and E2F1-responsive Gene Expression via Association with Host Cell Factor-1 (HCF-1). J Biol Chem 288: 17532–17543. 23629655 10.1074/jbc.M112.439729 PMC3682552

[26] Bhattacharya A, Tejero R, Montelione GT (2007) Evaluating protein structures determined by structural genomics consortia. Proteins 66: 778–795. 17186527 10.1002/prot.21165

[27] Huang YJ, Powers R, Montelione GT (2005) Protein NMR Recall, Precision, and F-measure scores (RPF scores): structure quality assessment measures based on information retrieval statistics. J Am Chem Soc 127: 1665–1674. 15701001 10.1021/ja047109h

[28] Koradi R, Billeter M, Wüthrich K (1996) MOLMOL: a program for display and analysis of macromolecular structures. J Mol Graphics 14: 51–55. 10.1016/0263-7855(96)00009-4 8744573

[29] Celniker G, Nimrod G, Ashkenazy H, Glaser F, Martz E, et al (2013) ConSurf: Using Evolutionary Data to Raise Testable Hypotheses about Protein Function. Isr J Chem 53: 199–206.

[30] Ali M, Rincon-Arano H, Zhao W, Rothbart SB, Tong Q, et al (2013) Molecular Basis for Chromatin Binding and Regulation of MLL5. Proc Natl Acad Sci U S A 110: 11296–11301. 23798402 10.1073/pnas.1310156110 PMC3710826

[31] Holm L, Sander C (1993) Protein structure comparison by alignment of distance matrices. J Mol Biol 233: 123–138. 8377180 10.1006/jmbi.1993.1489

[32] Sebastian S, Sreenivas P, Sambasivan R, Cheedipudi S, Kandalla P, et al (2009) MLL5, a trithorax homolog, indirectly regulates H3K4 methylation, represses cyclin A2 expression, and promotes myogenic differentiation. Proc Natl Acad Sci U S A 106: 4719–4724. 19264965 10.1073/pnas.0807136106 PMC2651835

[33] Nady N, Min J, Kareta MS, Chédin F, Arrowsmith CH (2008) A SPOT on the chromatin landscape? Histone peptide arrays as a tool for epigenetic research. Trends Biochem Sci 33: 305–313. 18538573 10.1016/j.tibs.2008.04.014

[34] Chakravarty S, Zeng L, Zhou M (2009) Structure and site-specific recognition of histone H3 by the PHD finger of human autoimmune regulator. Structure 17: 670–679. 19446523 10.1016/j.str.2009.02.017 PMC2923636

[35] Zhang Y, Reinberg D (2001) Transcription regulation by histone methylation: interplay between different covalent modifications of the core histone tails. Genes Dev 15: 2343–2360. 11562345 10.1101/gad.927301

[36] Collins RE, Northrop JP, Horton JR, Lee DY, Zhang X, et al (2008) The ankyrin repeats of G9a and GLP histone methyltransferases are mono- and dimethyllysine binding modules. Nat Struct Mol Biol 15: 245–250. 18264113 10.1038/nsmb.1384 PMC2586904

[37] Gutmanas A, Jarvoll P, Orekhov V, Billeter M (2002) Three-way decomposition of a complete 3D 15N-NOESY-HSQC. J Biomol NMR 24: 191–201. 12522307 10.1023/a:1021609314308

[38] Lemak A, Gutmanas A, Chitayat S, Karra M, Fares C, et al (2011) A novel strategy for NMR resonance assignment and protein structure determination. J Biomol NMR 49: 27–38. 21161328 10.1007/s10858-010-9458-0 PMC3715383

[39] Delaglio F, Grzesiek S, Vuister GW, Zhu G, Pfeifer J, et al (1995) NMRPipe: a multidimensional spectral processing system based on UNIX pipes. J Biomol NMR 6: 277–293. 8520220 10.1007/BF00197809

[40] Lemak A, Steren CA, Arrowsmith CH, Llinas M (2008) Sequence specific resonance assignment via Multicanonical Monte Carlo search using an ABACUS approach. J Biomol NMR 41: 29–41. 18458824 10.1007/s10858-008-9238-2

[41] Goddard TD, Kneller DG (2003) Sparky – NMR assignment and integration software. University of California, San Francisco.

[42] Cornilescu G, Delaglio F, Bax A (1999) Protein backbone angle restraints from searching a database for chemical shift and sequence homology. J Biomol NMR 13: 289–302. 10212987 10.1023/a:1008392405740

[43] Gunter P (2004) Automated NMR protein structure calculation with CYANA. Meth Mol Biol 278: 353–378. 10.1385/1-59259-809-9:353 15318003

[44] Brunger AT, Adams PD, Clore GM, DeLano WL, Gros P, et al (1998) Crysttallography & NMR systems: A new software suite for macromolecular structure determination. Acta Crystallographica Section D 54: 905–921. 10.1107/s0907444998003254 9757107

[45] Linge JP, Williams MA, Spronk AEM, Bonvin AMJJ, Nilges M (2003) Refinement of protein structures in explicit solvent. Proteins 50: 496–506. 12557191 10.1002/prot.10299

[46] Laskowski RA, MacArthur MW, Moss DS, Thornton JM (1993) PROCHECK: a program to check the stereochemical quality of protein structures. J Appl Cryst 26: 283–291.

[47] Davis IW, Leaver-Fay A, Chen VB, Block JN, Kapral GJ, et al (2007) MolProbity: all-atom contacts and structure validation for proteins and nucleic acids. Nucleic Acids Res 35: W375–W383. 17452350 10.1093/nar/gkm216 PMC1933162

