# Supplementary figures and images for: Solution NMR Structure and Histone Binding of the PHD Domain of Human MLL5
Source: PLoS One. 2013 Oct 9;8(10):e77020. doi: 10.1371/journal.pone.0077020 (PMC3793974; doi:10.1371/journal.pone.0077020)

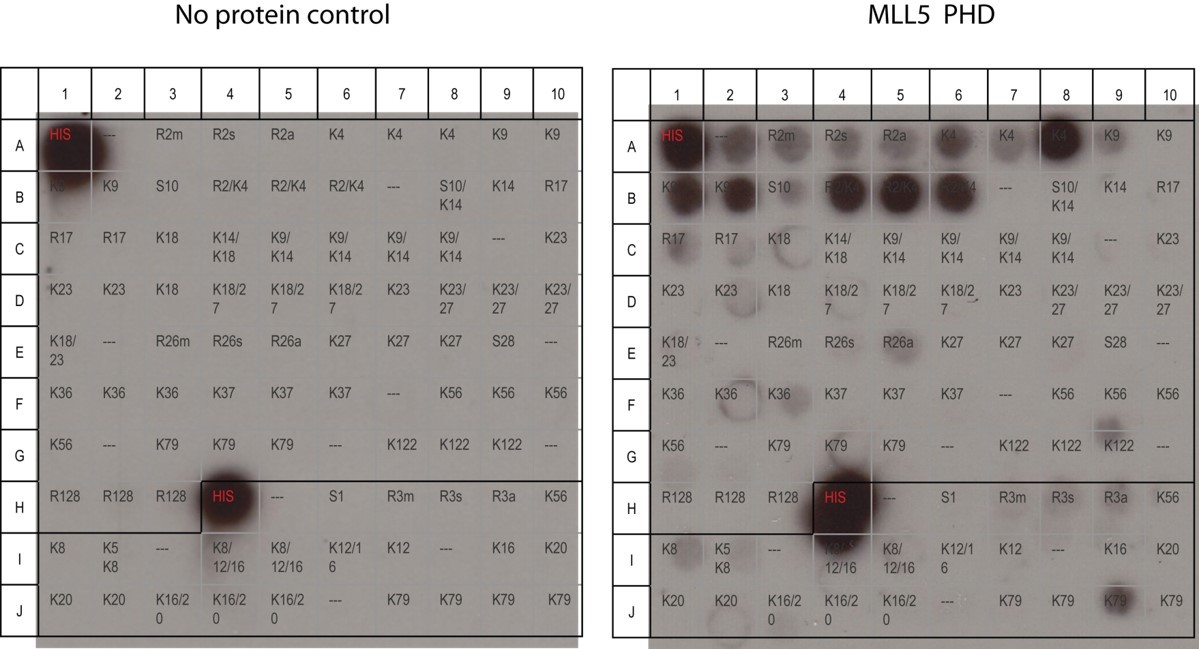

Supplement: Figure S1 — H3 histone tail peptide array bound with his-tagged MLL5PHD. Protein was detected using anti-His antibody. Left panel showed the no protein control, only the poly-His spot was detected by the anti-His antibody. Right panel showed the peptide spots where MLL5PHD proteins were bound. The letters on each grid highlight which residues on the H3 histone tail was modified. Actual peptide sequence on the array is shown in Supplementary Figure S2. (JPG) [file pone.0077020.s001.jpg]

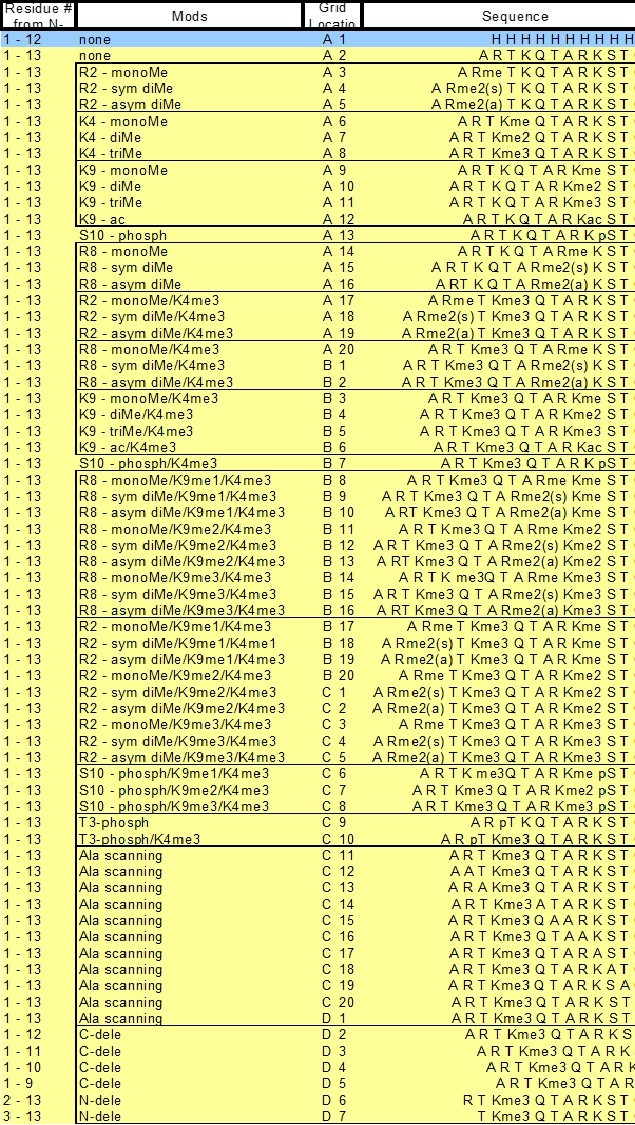

Supplement: Figure S2 — Peptide sequence of the peptide array in Figure S1. Grid location refers to Figure S1a. (JPG) [file pone.0077020.s002.jpg]
